# Supplementary material for: C5, A Cassaine Diterpenoid Amine, Induces Apoptosis via the Extrinsic Pathways in Human Lung Cancer Cells and Human Lymphoma Cells
Source: Int J Mol Sci. 2020 Feb 14;21(4):1298. doi: 10.3390/ijms21041298 (PMC7072863; doi:10.3390/ijms21041298)
Supplement: Supplementary file 1 [file ijms-21-01298-s001.pdf]

Supplementary Materials

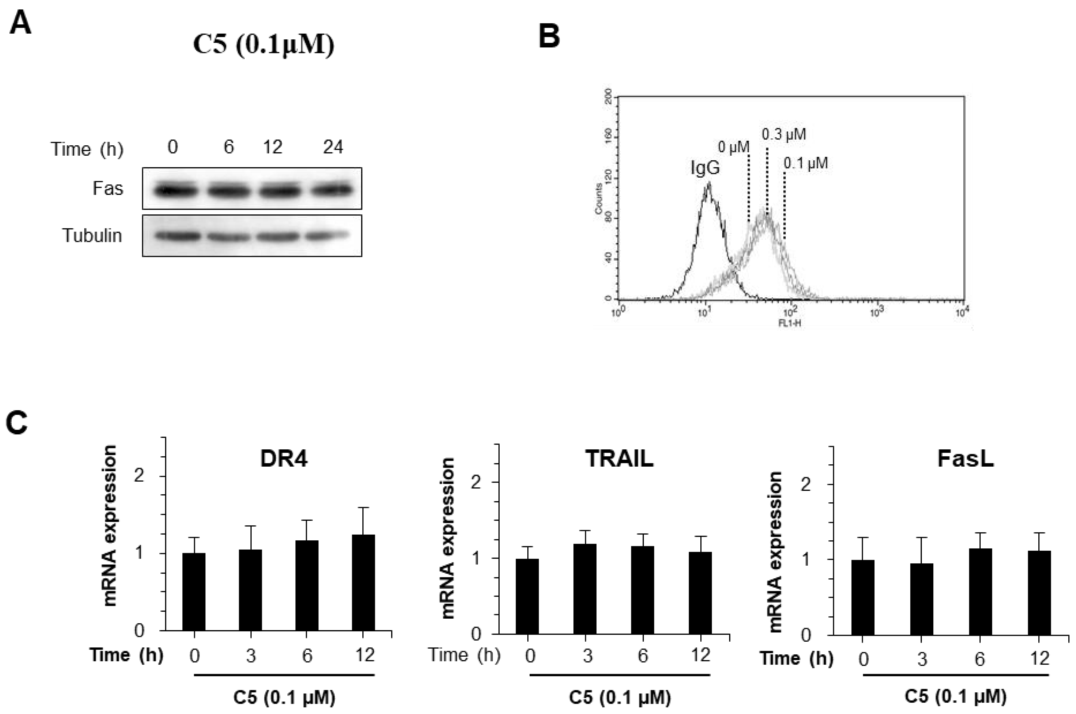

Figure S1. C5 does not regulates expression of death receptor and death ligand. (A) The expression level of the death-receptor for C5 treated as 0.1μM at the indicated time in Ramos cells. (B) Flow cytometry assay for measurment of death receptor expression in response to C5 at the indicated concentration in Ramos cells. (C) After treated with 0.1μM C5 at the indicated time in Ramos cells, mRNA level of death ligands measured by qRT-PCR.
